# Durability Indicators Comparison for SCC and CC in Tropical Coastal Environments

**DOI:** 10.3390/ma8041459

**Published:** 2015-03-27

**Authors:** Carlos Calado, Aires Camões, Eliana Monteiro, Paulo Helene, Béda Barkokébas

**Affiliations:** 1 CTAC, Department of Civil Engineering, University of Minho, Guimarães 4800-058, Portugal; E-Mail: aires@civil.uminho.pt; 2 Program of Civil Engineering, University of Pernambuco, Recife 50100-010, Brazil; E-Mails: eliana@poli.br (E.M.); beda.jr@upe.br (B.B.); 3 Department of Civil Engineering, University of São Paulo, PhD Engenharia, São Paulo 05508-900, Brazil; E-Mail: paulo.helene@concretophd.com.br

**Keywords:** self-compacting concrete (SCC), conventional vibrated concrete (CC), durability

## Abstract

Self-compacting concrete (SCC) demands more studies of durability at higher temperatures when subjected to more aggressive environments in comparison to the conventional vibrated concrete (CC). This work aims at presenting results of durability indicators of SCC and CC, having the same water/binder relations and constituents. The applied methodologies were electrical resistivity, diffusion of chloride ions and accelerated carbonation experiments, among others, such as microstructure study, scanning electron microscope and microtomography experiments. The tests were performed in a research laboratory and at a construction site of the Pernambuco Arena. The obtained results shows that the SCC presents an average electrical resistivity 11.4% higher than CC; the average chloride ions diffusion was 63.3% of the CC; the average accelerated carbonation penetration was 45.8% of the CC; and the average open porosity was 55.6% of the CC. As the results demonstrated, the SCC can be more durable than CC, which contributes to elucidate the aspects related to its durability and consequent prolonged life cycle.

## 1. Introduction

In structures, concrete is usually associated with steel rebar to form reinforced concrete or prestressed concrete. The structure will interact with the environment around it and, as a consequence, its materials are willing to develop reactions that may alter its initial conditions, not fulfilling the role that they were designed to perform, endangering, in this way, its durability.

According to Neville [1], concrete must be able to withstand the expected process of deterioration due to the surrounding environment. When that is achieved, it is possible to say that the concrete will be durable. However, undefined life cycle does not mean durability, and also it is not related to the ability to bear any mechanical or aggressive load on the concrete. Neville [1] recognizes that the main importance was always given to mechanical strength of concrete. However, nowadays it is assumed that strong concrete is a durable concrete, and both properties, mechanical strength and durability, must be considered explicitly in the design phase.

It is understood that, with the exception of mechanical damage, adverse influences on the durability involve the transportation of fluids, such as pure water or water carrying chloride ions, carbon dioxide and oxygen. These fluids, liquids and gases, can penetrate and move inside the concrete by different paths through the porous media, not only because of the fluidity, but also because of diffusion and absorption. The interconnected pores contribute to increase the permeability. The absorption occurs because of the capillarity effect in the concrete pores that are open to the environment; suction may only occur in a partially dry concrete, and no water can be absorbed in a completely dry or saturated concrete. On the other hand, diffusion occurs when the transportation of a gas or steam through concrete is the result of a concentration gradient and not from a pressure difference, like where carbon dioxide leads to the carbonation of the hydrated paste and oxygen allows the corrosion progress of the reinforcements involved in the concrete. Therefore, an impermeable and dense concrete will greatly reduce the entrance of aggressive agents in its interior, limiting corrosive attacks only on its surface. The water/cement ratio, temperature, level of hydration, cement compounds and mineral additions incorporation, capillary porosity, permeability, among other factors, influence the durability properties of the self-compacting concrete (SCC) and conventional vibrated concrete (CC) [2,3,4,5].

In Brazil, since 2003, with the new standard NBR 6118, *Design of structural concrete—Procedure* [6], technicians who design concrete structures are more concerned about the concept and the ways to obtain a higher durability in concrete construction sites.

Recife, a coastal city, capital of Pernambuco state, is considered the fifth largest metropolitan population in Brazil, with 4,046,845 citizens [7]. It combines harmful conditions for reinforced concrete structures, such as annual average high temperatures of 29.1 °C, relative humidity of 79.8%, pluviometric precipitation of 2417.6 mm, 2550.7 h sunny hours, besides marine atmosphere according to National Weather Institute (INMET) [8]. Thus, there is an adequate environment to increase the chloride diffusion rate and of contamination by carbonation in the concrete, among other factors that contribute to the deterioration of reinforced concrete elements. The amount of conventional concrete construction sites that does not reach the project expected service life tends to be higher if mitigating actions are not adopted in the steps of design, execution, and maintenance [9,10]. Another relevant aspect in Recife’s region was the occurrence of pathologies typical of an alkali-aggregate reaction [11].

De Schutter [2] verified that much is known about durability of CC, but there are still problems once a vibration operation is needed to promote the densification. Because of the self-compacting ability, SCC does not need any vibration and therefore will not be affected by these problems, although less is known about its durability. Therefore it is necessary to study the durability of SCC in comparison to CC in tropical coastal environments, which have more aggressive climates and higher temperatures [12].

The present work presents studies applied to hardened concrete, developed in research laboratories associated with studies applied to a construction site of the Pernambuco Arena. The Pernambuco Arena was constructed between 2011 and 2013 to receive the Confederations Cup in 2013 and the World Cup in 2014, both sponsored by FIFA. The Arena has a constructed area of 128,000.0 m^2^, capacity of 46,105 people, with an approximate total volume of applied concrete of 58,000.0 m^3^, being 40% (23,200.0 m^3^) SCC and 60% (34,800.0 m^3^) CC. The goal was to identify possible differences between SCC and CC, allowing establishing which one of the concretes presents higher durability indicators [13].

The literature presents some recent specific studies of durability and resistance applied to SCC and CC related to the effect of water/cement ratio, cement, binder composition variation, and especially addition of metakaolin, according to the authors of these studies [11,14,15,16]. The objective was to study the behavior of SCC and CC, comparing their performance to sulfate resistance, carbonation, porosity, electrical resistance, permeability and chloride penetration. The results showed that, in general, SCC presents better performance than CC. It is also possible to establish a more adequate composition of each concrete with determined resistance and durability characteristics demanded by mechanical requirements and environmental conditions [17,18,19,20,21].

## 2. Experimental Methodology

### 2.1. Concrete Composition for Specimen Molding

For the laboratory studies, two compositions of concrete were established: one for SCC (SCC_Lab) and another for CC (CC_Lab), with constituents usually found and applied to the selected region, in order to ensure similarity with the concretes used there. This similarity between the compositions of SCC_Lab and CC_Lab was considered on account of both the same water/binder and cement/fine ratios and the use of the same constituents in both compositions. In respect to the Pernambuco Arena construction sites, SCC (SCC_AP) and CC (CC_AP) compositions were chosen among the largest applied volume with similar characteristics [22].

In the laboratory, the following tests were performed on SCC_Lab and CC_Lab samples: compressive strength, electrical resistivity, diffusion of chloride ions, accelerated carbonation, open porosity, and water absorption by capillarity. Another aspect to be considered is the microstructure of SCC compared to CC. The absence of vibration, associated with the possible existence of a higher amount of paste in SCC, does cause modifications in the interfacial transition zone between aggregates and paste in comparison to CC. Thus, scanning electron microscopy (SEM) and microtomography (MT) techniques were used to study and compare the microstructure of both types of concrete [23,24,25,26].

The experiments developed at the construction site of the Pernambuco Arena were carried out with samples of SCC_AP and CC_AP. For the hardened concrete, the following experiments were performed: compressive strength, elasticity modulus, diffusion of chloride ions, open porosity and water absorption. In order to simulate as close as possible the conditions of the construction sites used, the samples of SCC_AP and CC_AP were divided in two types: those stored at the construction site in the same conditions as in the laboratory (SCC_AP-CP and CC_AP-CP); and those ones extracted as concrete cores from concrete elements deliberately produced for these tests and cast, cured and exposed to the same conditions as the concrete applied to the structural elements of the construction work (SCC_AP-TE and CC_AP-TE).

**Figure 1:**
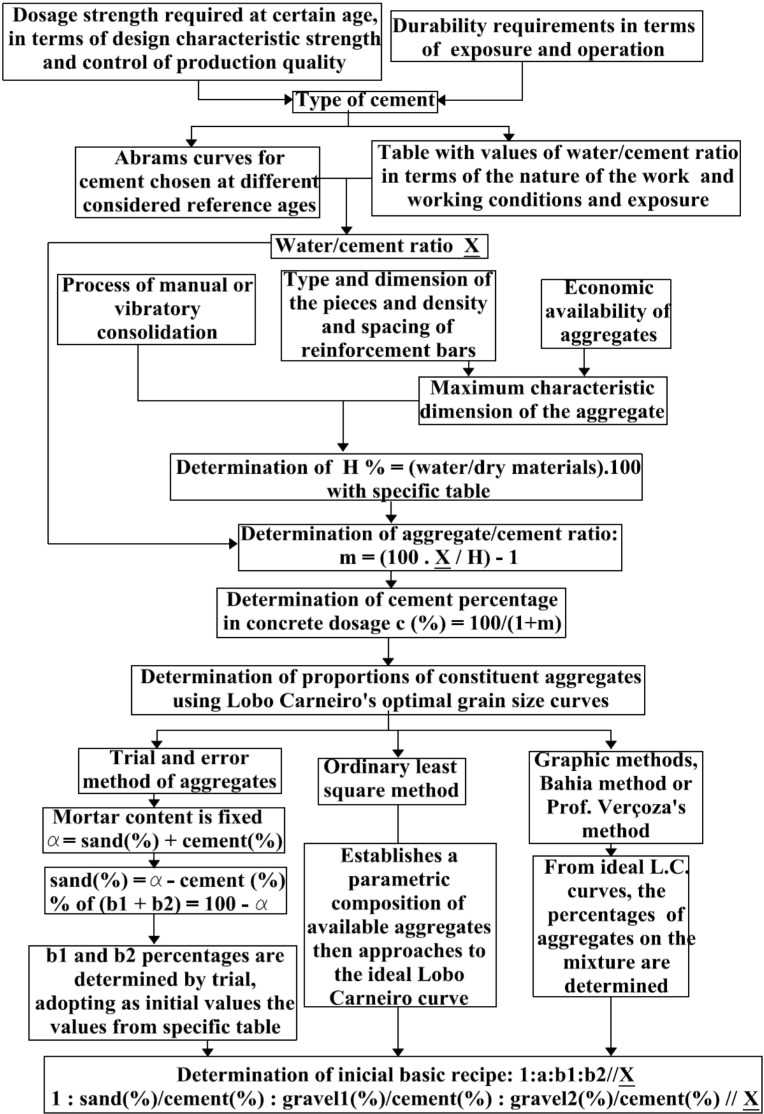
Simplified flowchart of the dosage method, known as INT/Lobo Carneiro [27].

The concrete mix-design methodology, known as INT and proposed by Lobo Carneiro [27,28], was used in this study. This method searches for an optimum particle size proportion of the selected aggregates, with the intent of obtaining the maximum compactness of the concrete mixture. The activities, variables and input information of the method may be summarized in this way: the concrete strength required at a certain age and the durability requirements lead to the selection of the type of cement; Abrams curves and water/cement ratio, as a function of workability, lead to an appropriate water/cement ratio; the densification process and maximum dimension of the aggregate lead to a water/dry materials ratio that, associated to a water/cement ratio, leads to an aggregate/cement ratio; then, a percentage of cement in the composition of the concrete and the determination of the proportions of the mixture aggregate components are determined by optimum particle size curves proposed by Lobo Carneiro (see Figure 1).

The SCC and CC compositions were chosen in order to ensure high similarity between them. Thus, the appropriate cement content in each composition was determined so that the same water/cement and cement/fine ratios were obtained. Thereby, for the laboratory tests, 419 kg/m^3^ and 416 kg/m^3^ of cement were used for SCC and CC, respectively. For the construction site of the Pernambuco Arena, 499 kg/m^3^ and 451 kg/m^3^ of cement were used for SCC and CC, respectively. Also, the other components used, such as aggregates, mineral additions and chemical admixtures, were the most commonly used in the region. Metakaolin was added to SCC_Lab and CC_Lab compositions used in the research laboratory, as it has been used as an alternative to avoid preventively deleterious reactions verified in the region, such as alkali-aggregated type according to [14,15,16].

### 2.2. Materials and Components Applied in the Concretes

For the research laboratory studies, used Portland cement CP-V ARI was used, with high initial resistance, which is equal to the CEM I 42.5 cement in Europe. For the studies in the Pernambuco Arena construction site, the selected compositions were the Portland compound cement with limestone filler CP-II F 32, which is equal, in Europe, to CEM II/A-L 32.5 cement. The chemical admixtures applied were: (a) a plasticizer with a high water reduction effect composed of sulfonated salt and carbohydrates in aqueous medium, with density of 1190 kg/m^3^ and pH of 5.5; and (b) a liquid superplasticizer of third generation normal grip, composed of polycarboxylates in aqueous medium, density of 1060 kg/m^3^ and pH of 5.0. The thin and coarse aggregates used were the ones available in the market, and the same ones were applied in the SCC_Lab, CC_Lab, SCC_AP, and CC_AP compositions. The coarse aggregate was crushed from granitic stone, and the thin aggregate was obtained from a quartz origin, extracted from riverbed deposits. The water used to prepare the compositions for the experiments in the laboratory were provided by Compesa, a local company of treated water for public consumption. The water for the tests in the Pernambuco Arena was provided by a drilled well at the location [29].

Table 1 presents the compositions of SCC and CC used in our experiments, for both studies in the research laboratory, SCC_Lab and CC_Lab, and in the Pernambuco Arena, SCC_AP and CC_AP. The amounts of each constituent are indicated per cubic meter of concrete, but it is worth mentioning that all of them are commonly employed regionally. The site concrete mixtures shown were used in the superstructure construction, where metakaolin is usually not applied locally. The other differences from laboratory mixtures result from the analysis of the present studies and site construction experience, which indicated the need for at least 90 minutes from output of the concrete plan to actual casting.

The workability of the fresh compositions is also presented in Table 1, where the obtained results of slump flow for SCC or slump for CC are shown.

**Table 1:** Compositions of SCC and CC.

Components	Unit	Laboratory concrete		Construction site concrete	
		SCC_Lab	CC_Lab	SCC_AP	CC_AP
Cement ^(1)^	kg/m^3^	419	416	499	451
Metakaolin	kg/m^3^	36	36	-	-
Sand	kg/m^3^	947	661	856	815
Aggregate 1 12.5 mm	kg/m^3^	227	-	-	-
Aggregate 2 19.1 mm	kg/m^3^	529	1028	830	917
Water	kg/m^3^	205	203	199	180
Superplasticizer	kg/m^3^	5	-	4.9	2.1
Plasticizer	kg/m^3^	4.20	2.60	3.40	4.40
water/binder		0.45	0.45	0.40	0.40
cement/fine		0.92	0.92	1.00	1.00
Slump flow or Slump	mm	700	416	499	451

Note: ^(1)^ CEM I 42.5 and CEM II/A-L 32.5 equivalent cements used in the research laboratory studies and in the Pernambuco Arena construction work, respectively.

### 2.3. Types of Specimens Used in the Experiments in the Pernambuco Arena Construction Site

**Figure 2:**
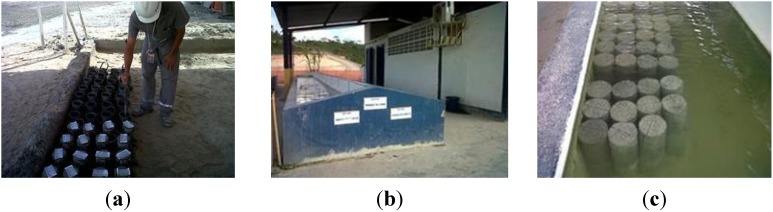
Cure of the samples at laboratory conditions. (**a**) specimen’s casting; (**b**) curing tank; (**c**) specimens immersed in the curing tank.

The experiments were carried out with two types of samples collected from the concrete that would be used in the structural elements of the construction work. The first type of sample was specimens molded and stored in laboratory conditions at the construction site (named SCC_AP-CP and CC_AP-CP), according to EN 12390-2:2009 Testing hardened concrete—Part 2: Making and curing specimens for strength tests, as presented in Figure 2a–c. The curing process was water immersion. The curing process was done during the coldest months of the year, *i.e.*, May, June and July, with maximum, average, and minimum temperatures equal to 29 °C, 25 °C and 21 °C, respectively. The average relative humidity was 80%.

**Figure 3:**
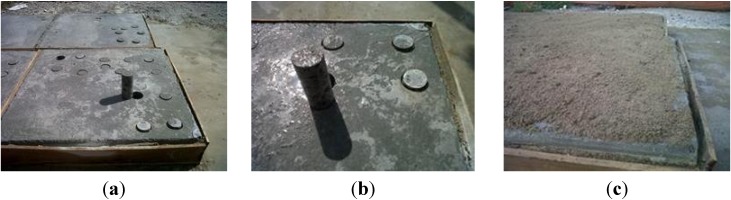
Cure of the specimens at local atmospheric conditions. (**a**) concrete core; (**b**) detail of concrete core; (**c**) concrete block.

The second type of samples was obtained by the extraction of specimens from molded and cured concrete elements (named SCC_AP-TE and CC_AP-TE) at the same conditions as the one applied in the construction work, at the same local environment, as can be seen in Figure 3.

From the group of compositions really used in the Pernambuco Arena, two of the most applied ones were selected: one SCC (SCC_AP) and one CC (CC_AP), which together represent 55% of the total amount of concrete applied during May, June and July, 2012. Both compositions may be considered similar, since they have the same water/cement ratio (W/C = 0.40) and the same constituents, despite different proportions. SCC_AP composition was applied to walls and columns, and CC_AP composition was applied in beams.

### 2.4. Compressive Strength and Elasticity Modulus: Experiments Performed in the Research Laboratory and at the Construction Site of the Pernambuco Arena

Compressive strength was determined through tests realized in specimens of hardened concrete at 3, 7, 14, 28, 56 and 90 days of age, according to EN 12390-3:2009 Testing hardened concrete—Part 3: Compressive strength of test specimens. For these tests, six specimens of cylindrical geometry, with 100 mm of diameter and 200 mm of height were prepared. For each age, three specimens were SCC ones and the rest were casted with the tested CC. Before starting the experiments, the bases of the specimens were adjusted according to EN 12390-2:2009. In order to determine the static elastic modulus in compression, at the construction site of the Pernambuco Arena, tests were performed according to the EN 12390-13:2013 Testing hardened concrete to determine the secant modulus of elasticity in compression, for SCC_AP and CC_AP samples.

### 2.5. Electrical Resistivity of Concrete: Experiments Performed in the Research Laboratory

**Figure 4:**
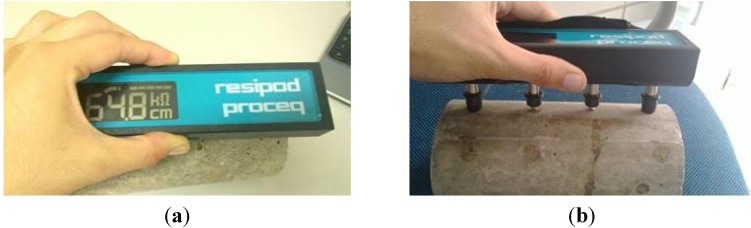
Visualization and measurement of the electrical resistivity. (**a**) electrical resistivity apparatus; (**b**) measuring electrical resistivity.

Electrical resistivity means the relative capacity of a medium to conduct electric current. This way, after the dissolution or breakage of the passive layer, concrete resistivity starts to be one of the factors that affect the speed of corrosion. The measurement method is nondestructive and indicates a superficial resistivity of the concrete that depends preponderantly on the amount of electrolytes, water and salts contained in the interstices of the concrete [30]. The experiment of electrical resistivity was performed according to RILEM TC154-EMC [31]. For this experiment, six specimens were prepared: three of self-compacting concrete (SCC_Lab) and three of conventional vibrated concrete (CC_Lab). The specimens used had dimensions of Φ100 mm × 200 mm. The electrical resistivity readings were performed at the ages of 3, 7, 28, 56 and 90 days. Figure 4 shows the equipment being used to measure the electrical resistivity of the specimens.

The criteria used in the analysis and classification of the probable corrosion rate, as a function of the electrical resistivity of the concrete, are presented in Table 2, according to CEB 192 [32].

**Table 2:** Probability of corrosion as a function of the electrical resistivity CEB 192 [32].

Concrete resistivity ρ (kΩcm)	Probable corrosion rate
>20	Despicable
Between 10 and 20	Low
Between 5 and 10	High
<5	Very high

### 2.6. Chloride Ions Diffusion: Experiments Performed in the Research Laboratory and at the Construction Site of the Pernambuco Arena

Evaluating the chloride diffusion through the cement paste is important; as these ions may start the corrosion process of the reinforcements, one of the pathologic manifestations most commonly identified in reinforced concrete structures. It is estimated that the diffusion rate varies linearly with temperature and is inversely proportional to concrete age (ABCP, 2012). At the ABCP (Brazilian Association of Portland Cement) laboratory, tests were performed to determine the chloride ions diffusion in specimens of 28 and 90 days of age, according to ASTM C1202 [33]. For tests performed in the laboratory at each age, six specimens were used: three of self-compacting concrete (SCC) and three of conventional vibrated concrete (CC). At the Pernambuco Arena, tests were carried out in samples with 60 days of age. The total charge measured in Coulombs (C) is related to the concrete resistance to chloride ions penetration. The obtained results are semi-quantitative, in view of the amplitude of the classificatory ranges presented at ASTM C1202 [33], as shown in Table 3.

**Table 3:** Classification criteria for chloride diffusion, ASTM C1202 [33].

Charge flow (coulombs)	Chloride ions penetration
>4000	High
2000–4000	Moderate
1000–2000	Low
100–1000	Very low
<100	Negligible

### 2.7. Accelerated Carbonation: Experiments Performed in the Research Laboratory

The accelerated carbonation tests are systematically used in the corrosion evaluation of the reinforcement with satisfying results. The procedure consists of using a carbonation chamber in which CO_2_ is inserted at a higher content than those found in the atmosphere. Several authors have been studying natural and accelerated carbonation and its influence in the concrete durability. Nunes *et al.* [34] researched concretes with partial replacement of cement by 5%, 10%, 20% and 30% of rice husk ash resulted in an increase in the carbonation coefficient compared to the reference samples. Moreira *et al.* [35] studied the influence of environments with high temperature on concrete carbonation depth submitted to different types of cure and did not observe a significant difference between the carbonation depths in specimens submitted to wetting twice a day for three to seven days of curing period. The studies concluded that simple wetting cure shows no benefit in terms of carbonated depth. Possan *et al.* [36] in her case study of the *Itaipú concrete dam* confirmed the influence of exposure environment and moisture on the carbonation depth test. The researchers observed that the influence of rain protection is more important where carbonation is higher in external unprotected environments, protected external environments and indoors environments. These findings are similar to results found by Helene *et al.* [37], which deals with a case study of a port structure in Recife where carbonation depth testing was applied to eight columns covering all building facades. Only the west facade column was not carbonated, because this was an external column protected from the rain compared to the other seven columns unprotected, where carbonation front had reached the reinforcement.

**Figure 5:**
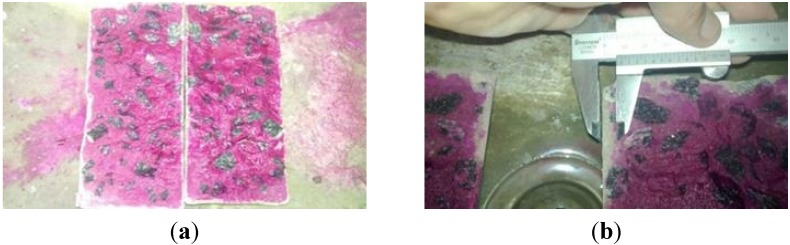
View and measurement of the carbonation front. (**a**) phenolphthalein aspersed specimen; (**b**) measuring carbonation depth.

The accelerated carbonation test was performed in samples of hardened concrete with ages of 28 and 90 days, following the recommendations of RILEM TC056-CPC-18 [38]. For this test, sixteen cylindrical specimens (100 mm diameter and 200 mm high) were prepared for each age: eight of self-compacting concrete (SCC_Lab) and eight of conventional concrete (CC_Lab). The specimens were stored in laboratory conditions, with relative humidity of 70% ± 10% and an average temperature of 29 °C, until the execution of the test after attaining constant mass; they were kept at humidity and temperature conditions similar to the carbonation chamber. The specimens used in the accelerated carbonation process were conditioned in a carbonation chamber with a CO_2_ content of 7.5% ± 2.5%, inside relative humidity of 70% ± 10% and an average temperature of 29 °C. The exposure time in the carbonation chamber was 22 calendar days. This period was considered enough to compare the results for SCC and CC samples. After 22 days of the experiment, the specimens were removed from the chamber and ruptured lengthwise. Next, a solution of 0.1% phenolphthalein was sprayed on the recently exposed surfaces, and then the eight most advanced points of the carbonation front, as shown in Figure 5, were measured.

### 2.8. Water Absorption by Capillarity: Experiments Performed in the Research Laboratory and at the Construction Site of the Pernambuco Arena

The determination of water absorption by capillarity on hardened concrete was performed, according to ASTM C1585-13:2013 Standard Test Method for Measurement of Rate of Absorption of Water by Hydraulic-Cement Concretes. For this test, six specimens were prepared for each age: three of self-compacting concrete (SCC) and three of conventional concrete (CC). The adopted ages for the laboratory experiments were 28 and 90 days. For the construction site of the Pernambuco Arena, the specimens were tested at an age of 35. Water absorption by capillarity must be expressed in g/cm^2^ and calculated by the ratio between mass increase and the cross sectional area of the specimen surface in contact with water, according to the following equation:

(1) $$C=\frac{\left(A-B\right)}{S}$$

where *C* = capillarity water absorption, in g/cm^2^; *A* = mass of a specimen that remains with one of its faces in contact with water during a specified period of time, in g; *B* = mass of the dry specimen, as soon as it reaches the temperature of 2 ± 23 °C, in g; *S* = cross sectional area, in cm^2^.

### 2.9. Open Porosity: Experiments Performed in the Research Laboratory and at the Construction Site of the Pernambuco Arena

The test for calculating open porosity in hardened concrete was performed according to ASTM C642-13:2013 Standard Test Method for Density, Absorption, and Voids in Hardened Concrete. The ages considered for the tests were 28 and 90 days, at the research laboratory, and 35 days, at the construction site of the Pernambuco Arena for specimens molded there. From ABNT, some definitions are: water absorption by immersion (A) = process in which water flows and may occupy the permeable pores of a porous solid body—therefore, this is the mass increase owing to this water penetration, in relation to its mass in dry state; void rate (Iv) = ratio between the volume of permeable pores and the total volume of the specimen; density of the dry specimen (ρs) = ratio between the mass of the dry material and the total volume of the specimen, including permeable and impermeable pores; density of the saturated specimen (ρsat) = ratio between the mass of the saturated material and the total volume of the specimens, including permeable and impermeable pores; and real density (ρr) = ratio between the mass of the dry material and its volume, excluding permeable pores. For this experiments, six specimens were used: three of self-compacting concrete (SCC) and three of conventional concrete (CC). The results were obtained by the following equations:

A (absorption) = [(msat − ms)/ms] × 100
(2)

Iv (void rate) = [(msat − ms)/(msat – mi)] × 100
(3)

ρs (density of the dry specimen) = ms/(msat – mi)
(4)

where msat = saturated mass; mi = saturated mass immersed in water after boiling; and ms = dry mass, determined in specimen after drying in heater for 72 h.

### 2.10. Scanning Electron Microscopy of Concrete–Interfacial Transition Zone Aggregate-Paste: Experiments Performed in the Research Laboratory

The scanning electron microscopy (SEM) experiments were performed with samples (SCC_Lab and CC_Lab) of the same batch. The microscope JSM T300 manufactured by Japan Electron Optics Laboratory Co. Ltd. established in Mitaka, Tokyo, with maximum magnification of 200,000.0 times and resolution of 6 nm (60 Å), operating with secondary electrons and equipped with dispersive energy spectrometer, with a Si/Li detector with a window of Be. The experiments used fragmented samples, metalized with gold, observed under the following analytical conditions: (1) Tension of 15 kV and (2) Current of 0.2 mA. Based on stereoscopic microscope observations, concrete fragments were separated in order to find portions more representative of the general texture of concretes and in particular of aggregate-paste interface. After selection, these samples were glued to a brass sample holder with graphite glue and metalized in an Edwards Metalizer, model S150B, with a film of gold.

**Figure 6:**
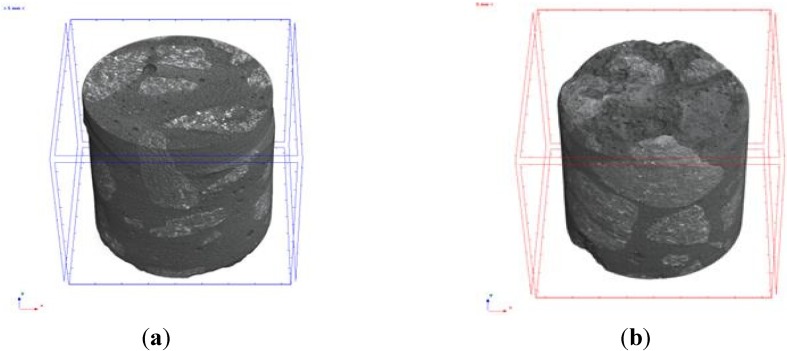
(**a**) Microtomography image of SCC samples and (**b**) of CC samples.

The microtomography (MT) tests were carried out with samples from the same batch using micro tomography [39], with X-ray fonts equal to 40–130 kV, 8 W, <5 µm local size. Initially, from the SCC_Lab and CC_Lab samples, it defined a volume of inspection (VOI), in order to establish the pore volume and its relationship with VOI. It also needed to establish the coefficient of attenuation to obtain the density profile. It is highlighted that, when establishing the relationship between the pore volume and VOI, such as the density profile, for SCC and CC samples, the priority was to compare results for both concretes and to identify which one presented higher durability conditions, namely, lower volume of pores. Figure 6 presents the microtomography images of the SCC and CC samples, respectively.

## 3. Results and Discussion

For the research laboratory studies (SCC_Lab and CC_Lab) and for the construction site of the Pernambuco Arena (SCC_AP and CC_AP), the specimens were molded at room temperature, 32 *°*C, with the goal of representing the real conditions of the region studied herein. Thus, if the results of the performed tests show that the durability of SCC is compatible or even higher than CC, its utilization may represent an advantageous option to be applied in structures constructed in the region.

### 3.1. Compressive Strength and Elasticity Modulus

Table 4 presents the average results of the compressive strength for the research laboratory concretes.

**Table 4:** Results of the compressive strength (f_cm_) for the research laboratory concretes.

Age (days)	f_cm_ (MPa)	
	SCC_Lab	CC_Lab
3	33.98 ± 1.71	32.32 ± 1.33
7	36.19 ± 4.87	33.03 ± 2.70
14	44.69 ± 1.31	40.39 ± 0.90
28	45.86 ± 2.33	42.69 ± 0.25
56	45.36 ± 2.38	43.28 ± 1.55
90	54.44 ± 2.57	43.80 ± 0.52

Analyzing Table 1, it is noted that the compositions used for SCC and CC are very similar; practically the same cement amount (419 kg/m^3^ for SCC and 416 kg/m^3^ for CC) and the same content of metakaolin (36 kg/m^3^), water/binder (0.45), and cement/fine (0.92). Because of this fact, very close compressive strengths for SCC and CC were expected. The main difference between both concretes was: 0.58% of plasticizer/binder for CC and 0.92% of plasticizer/binder plus 1.10% of superplasticizer/binder for SCC. Therefore, the hypothesis can be established that SCC achieves better packing, because of self-compactability that reduces the porosity, enhances the interfacial transition zone between the binder paste and the aggregates, and improves the strength in SCC mixtures [40,41,42]. Desnerck [43], based on a database with over 250 results of published papers, compared the 28 days compressive strength (f_c,28_) for SCC and CC and found values 10% higher for SCC, related to its higher microstructure density. Vita *et al.* [44], studying cracking of SCC at initial ages, obtained a better mechanical performance and a higher durability of SCC, because of its resistance to segregation and its fluidity eliminate macro defects, air bubbles and concreting failures. It is understood that these defects are directly responsible for losses in the mechanical and durability performance of the concrete structure.

Table 5 presents experimental average results of the compressive strength and elasticity modulus performed at the research laboratory and at the construction site of the Pernambuco Arena, in concretes at an age of 33 days.

It is verified that the average compressive strength of molded specimens in comparison with the cores was 1.2% higher for SCC and 3.6% for CC, which indicates a possible improvement of the stored concrete in laboratory conditions. However, the small difference only works as an indicative. For the average elasticity modulus of SCC, the cores were 3.1% higher than the specimens, contrary to the CC, whose average elasticity modulus of the molded specimens was 3% higher than the cores. So, the results are compatible with non-significant differences, as they are in the error range of the experiments.

**Table 5:** Compressive strength (f_cm_) and modulus of elasticity (E_cm_) for the Pernambuco Arena.

Composition	fcm (MPa)	Ecm (GPa)
SCC_AP-CP	62.58 ± 1.72	41.10 ± 0.87
SCC_AP-TE	63.14 ± 3.22	39.90 ± 1.17
CC_AP-CP	57.42 ± 1.69	38.20 ± 1.31
CC_AP-TE	56.73 ± 3.65	39.40 ± 2.32

### 3.2. Electrical Resistivity Experiment in the Research Laboratory

**Figure 7:**
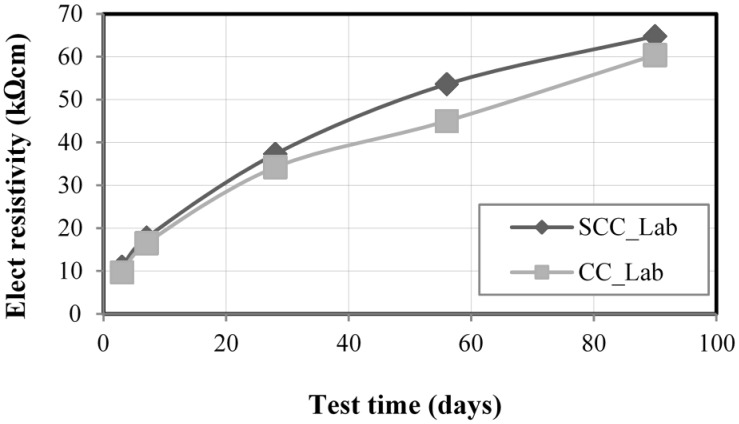
Electrical resistivity test results.

Figure 7 presents the average results of the electrical resistivity (in kΩcm) experiments, considering three measurements for each age and each concrete, for SCC_Lab and CC_Lab samples, at ages of 3, 7, 28, 56 and 90 days. At 28 and 90 days, SCC presented higher resistivity (9.1% and 7.3%, respectively) than CC. A slightly better performance of SCC can be explained by its higher hydration when comparing to CC. However, considering Table 2 that presents the corrosion probability as a function of the electric resistivity of the concrete based on CEB 192 [32], it is verified that SCC and CC, at both 28 days and 90 days, presented negligible probability of corrosion, despite a little higher resistivity of SCC in relation to CC. Therefore, it was found that, considering electric resistivity, SCC_Lab samples presented better performance than the CC_Lab ones, as the higher the electric resistivity of the concrete, the smaller the probability of having corrosion in the reinforced concretes. The increase of the electric resistivity as a function of age for both SCC and CC may be explained by a higher humidity inside concrete pores at lower ages.

### 3.3. Accelerated Carbonation Experiments for Research Laboratory

**Figure 8:**
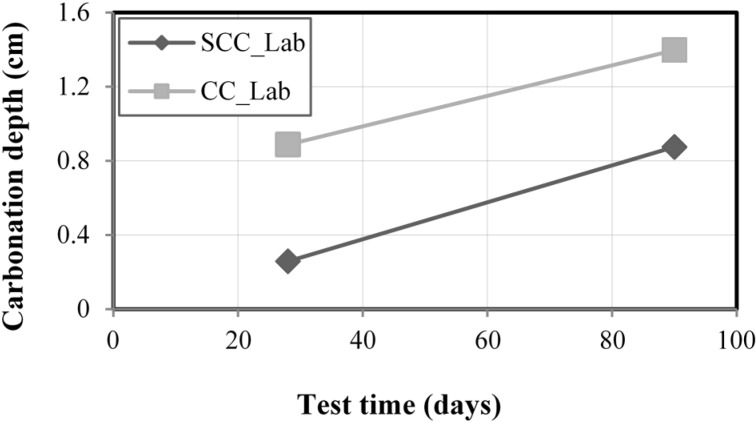
Accelerated carbonation test results.

Figure 8 shows the results for the carbonation depth in concrete based on the accelerated carbonating experiments performed in SCC_Lab and CC_Lab samples, at 28 and 90 days. The results are average values for each group of four samples. Coherence in the results obtained was noted. For SCC and CC, the carbonating front was higher in 90 days than in 28 days. As commented previously for the analysis of the electric resistivity, a higher humidity for SCC and CC samples at 28 days was verified. Based on this fact, it is possible to say that the carbonation is less probable in concretes with higher humidity, because the pores are filled with water, hindering the entrance of CO_2_. So, the statement that the carbonation front for SCC and CC was superior at 90 days can be explained by the humidity of the concrete. It is known that for humidity around 50% to 75%, there is more penetration of CO_2_ according to BRE DIGEST 263 [45]. On the other hand, the CC carbonation front values obtained were higher, on average, than SCC values, for 28 days (242%) and 90 days (59%). These results are also better than the ones found by Hartmann and Helene [46] for conventional concrete (33 MPa), whose carbonation depth was 2.8 cm, when subjected to accelerated carbonation process at 25 °C, relative humidity of 65% and CO_2_ of 5%. Thus, it was noticed that, regarding accelerated carbonation, SCC_Lab samples presented better performance than CC_Lab samples, as the higher the carbonation front of the concrete, the smaller the durability of concrete.

### 3.4. Chloride Ions Diffusion Experiments from the Research Laboratory and from the Construction Site of the Pernambuco Arena

**Figure 9:**
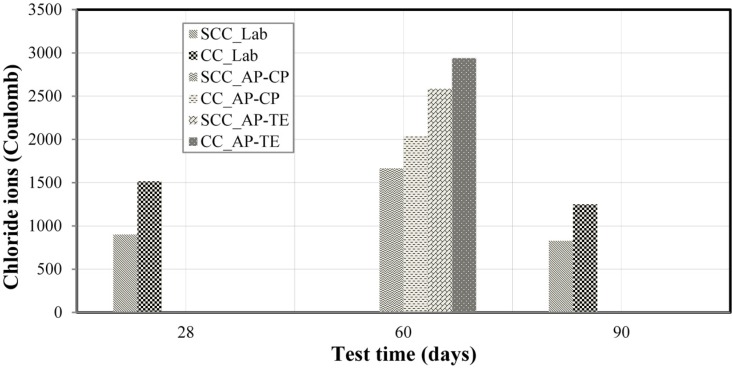
Results of chloride ions diffusion.

Figure 9 presents the results of the chloride diffusion coefficients obtained from the diffusion experiments in the non-steady state performed in the research laboratory for SCC_Lab and CC_Lab samples at 28 and 90 days. The result of the chloride diffusion coefficients for molded specimens and cores at 60 days for the construction site of the Pernambuco Arena is also presented. All values represent the average between the measurements for each sample studied and the result (C) represents the average charge flow in Coulombs.

With respect to the results of the experiments performed in the research laboratory (LP) shown in Figure 9, at 28 days, CC presented average charge flow in Coulombs 68.6% higher than SCC, while at 90 days this percentage was 51.0%. The higher the charge flow in Coulombs, the higher the penetration of chloride ions, which causes a decrease in the concrete durability and its reinforcements. The better performance of the SCC_Lab samples with respect to CC_Lab ones, despite the same water/binder ratio (0.45), can be explained because SCC samples are better sealed, internally, than CC ones. Based on ASTM C 1202 [33], the results showed very low chloride ions diffusion for SCC and low for CC, for both 28 and 90 days. The results for chloride ions penetration by diffusion experiments, obtained by Hartmann and Helene [46], were 43 C for 125 MPa concrete, and 8000 C for a 33 MPa concrete. Thus, we can affirm that SCC showed excellent performance.

For the experiments performed at the construction site of the Pernambuco Arena, it is verified that molded specimens and cores presented higher chloride ions diffusion for SCC and CC, even at 60 days, compared to the results at 28 days for SCC and CC from the research laboratory experiments (see Figure 9). The possible differences to be highlighted between the compositions used are: water/binder ratio of 0.45, CEM I 42.5 cement and addition of metakaolin, for the research laboratory experiments; water/cement ratio of 0.40 and CEM II/A-L 32.5 composed cement with limestone filler, for the experiments at the construction site of the Pernambuco Arena. It is observed that, possibly, the addition of metakaolin contributed to the reduction of chloride ions diffusion, which leads to an improvement in its durability.

### 3.5. Open Porosity for the Research Laboratory and for the Construction Site of the Pernambuco Arena

**Figure 10:**
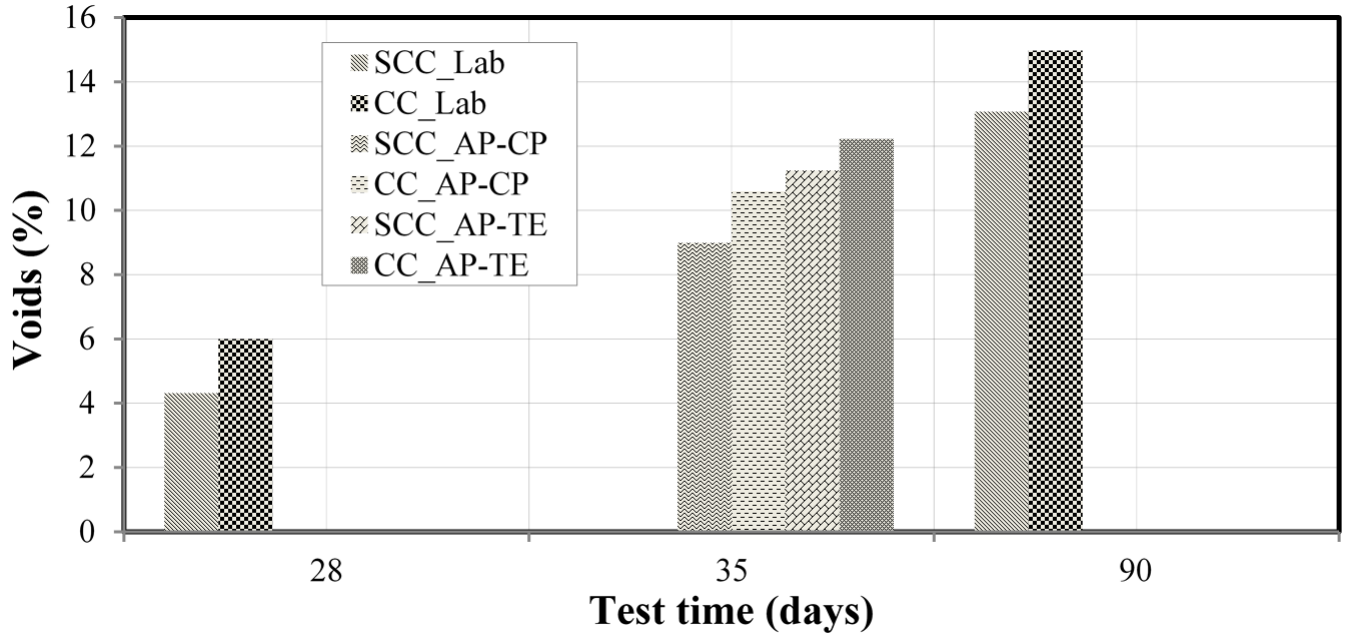
Results of void rate.

In Figure 10, one can see the results of the void ratio calculation experiments performed in research laboratory, for SCC and CC samples, at ages of 28 and 90 days, such as in the construction site of the Pernambuco Arena, applied in molded and core specimens, at the age of 35 days. The average measurements of each of the samples experimented were considered, and the results are shown in percentage (%). For the samples aged 28 days and 90 days, the void ratios obtained were higher for the CC in relation to the SCC, to the order of 39% at 28 days and 15% at 90 days. The results found were better than the results obtained by Helene [46] in the experiments with conventional concrete of 33 MPa compressive strength, where the obtained results of 5.8% of absorption after immersion and boiling and void ratios, after saturation and boiling, of 15.1%.

### 3.6. Capillarity Water Absorption Experiment in Research Laboratory and at the Construction Site of the Pernambuco Arena

**Figure 11:**
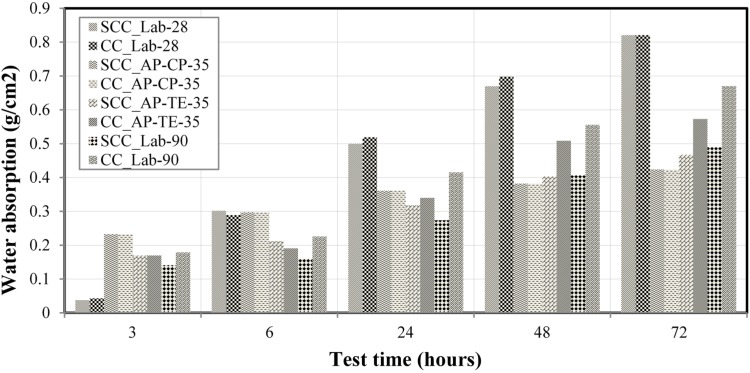
Results of capillarity water absorption in the performed experiments.

In Figure 11, the results of the capillarity water absorption experiments performed in research laboratory is presented in samples of SCC and CC at ages of 28 and 90 days and in the construction site of the Pernambuco Arena, applied in molded and cored specimens of 35 days. The average of the measurements in each of the experimented samples was considered, and the results of water absorption are shown in g/cm^2^. In this case: LP 28 means experiments performed in the research laboratory with 28 days of age samples, and LP 90 with 90 days; CP 35 means experiments performed in the construction site of the Pernambuco Arena with molded specimens of 35 days; and TE 35 with cored specimens at 35 days of age. In the research laboratory trials, it was verified that the results for 28 days were approximately equal for both SCC and CC, as well as in the results of 90 days, which did not present significant differences between SCC and CC. Monteiro [47] also observed in his experiments of capillary absorption in three kinds of cement studied no significant difference for the specimens tested at different ages. This is also concluded from analyzing the results presented in the same figure of the experiments performed in the construction site of the Pernambuco Arena with molded and cored specimens, at age of 35 days, using distinct compositions from the ones applied in the research laboratory.

### 3.7. Scanning Electron Microscopy and Microtomography Experiments at Research Laboratory

**Figure 12:**
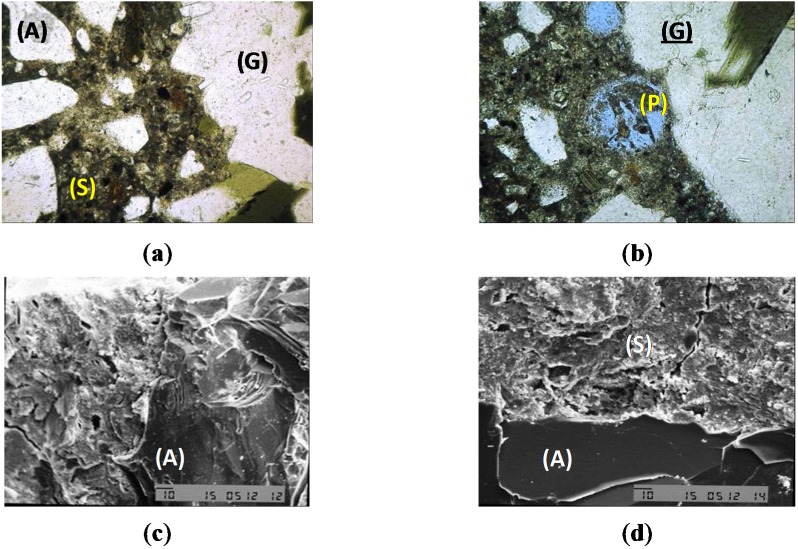
(**a**) SCC_Lab optical microscope image; (**b**) CC_Lab optical microscope image; (**c**) SCC_Lab SEM image; (**d**) CC_Lab SEM image.

Figure 12a presents the images of the analysis of the SCC_Lab sample though a light microscope, highlighting details of contact of the mortar phase with the great aggregate (G), the paste (S) and sand grains (A). Figure 12b presents the images of the CC_Lab sample analysis through an optical microscope, highlighting the aspect of a pore in the mortar (P) that develops itself in the edge of the great aggregate (G). Figure 12c presents the images of the analysis of the CC_Lab through SEM, highlighting the contact of the paste with sand grains (A). Figure 12d presents the images of the analysis of the CC_Lab sample through SEM, highlighting the contact of the paste with sand grains (A), where the higher porosity of the paste (S) can be observed.

**Figure 13:**
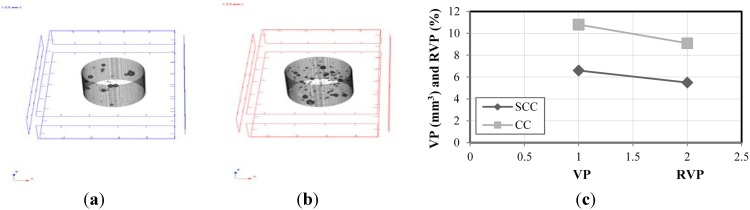
Results of the microtomography (MT) experiments. (**a**) SCC_Lab MT image; (**b**) CC_Lab MT image; (**c**) pore volume and total volume plot ratio.

Figure 13a,b presents the relationship between pore volume and volume of the samples of the SCC_Lab and CC_Lab, respectively, obtained through microtomography (MT) images. Figure 13c presents the results of pore volume (VP) (mm^3^) and the relation between pore volume and quantified volume of the samples (RVP) (%) of SCC and CC.

Based on the results obtained, it is verified that CC_Lab porosity was 65% higher than the SCC_Lab. Professor Geert De Schutter [26], developed research concerning durability of SCC *vs.* CC using micro-tomography (MT) was approached. In his study, the composition of SCC in relation to CC had an addition of limestone filler with an increase of the paste volume and reduction of the granular skeleton volume. The obtained result was that the porosity of CC, using the CC paste, was 74% superior to SCC using the limestone filler paste. Thus, the coherence of both studies seems to be verified.

### 3.8. Summary of Experimental Results

Table 6 presents a summary of all durability results, making it easier to compare the two types of concrete.

**Table 6:** Summary of results of durability experiments.

Experiment	Unit	Place of the Studies	Age		SCC	CC
Electrical resistivity	ρ(kΩcm)	RL ^(1)^	03 days		11.0	9.7
			07 days		17.8	16.5
			28 days		37.3	34.2
			56 days		53.6	45.0
			90 days		64.8	60.4
Diffusion of chloride ions	(C)	RL ^(1)^	28 days		900	1517
			90 days		828	1250
		PA-BP ^(2)^	60 days		1665	2040
		PA-ET ^(3)^	60 days		2585	2940
Accelerated carbonation	(cm)	RL ^(1)^	28 days		0.26	0.89
			90 days		0.88	1.40
Water absorption by capillarity	(g/cm^2^)	RL ^(1)^	28 days	3 hours	0.038	0.043
				6 hours	0.302	0.289
				24 hours	0.500	0.519
				48 hours	0.670	0.698
				72 hours	0.821	0.821
			90 days	3 hours	0.141	0.179
				6 hours	0.160	0.226
				24 hours	0.274	0.415
				48 hours	0.406	0.556
				72 hours	0.490	0.670
		PA-BP ^(2)^	35 days	3 hours	0.233	0.233
				6 hours	0.297	0.297
				24 hours	0.361	0.382
				48 hours	0.382	0.403
				72 hours	0.424	0.446
Water absorption by capillarity	(g/cm^2^)	PA-ET ^(3)^	35 days	3 hours	0.170	0.170
				6 hours	0.212	0.191
				24 hours	0.318	0.340
				48 hours	0.403	0.509
				72 hours	0.467	0.573
Voids	(%)	RL ^(1)^	28 days		4.31	6.00
			90 days		13.07	14.97
		PA-BP ^(2)^	35 days		8.99	10.59
		PA-ET ^(3)^	35 days		11.24	12.22
Microtomo graphy	(mm^3^)		Pore volume		6.60	10.80
	(%)		Pore Relation/VOI		5.50	9.10

Notes: ^(1)^ RL = Research Laboratory Experiments; ^(2)^ PA-BP = Pernambuco Arena Experiments in Bodies-of-Proof; ^(3)^ PA-ET = Pernambuco Arena Experiments Testimonials.

It is interesting to observe that self-compacting concrete presented better results in comparison to the conventional vibrated concrete demonstrating that besides the advantage in its application in a fresh state at a construction site due to its self-compacting quality, it presented good results in relation to the selected durability indicators of hardened concrete, demonstrating to be a better option in environments of higher aggressiveness.

## 4. Conclusions

Through applied methodology, it was possible to compare two types of concrete, self-compacting and conventional vibrated ones. The comparison was focused on the results of durability indicators against aggressive agents, besides compressive strength and elasticity module. The tests were performed in a research laboratory and at the construction site of Pernambuco Arena. In the construction site, two types of samples were established: molded specimens stored in laboratory conditions and cores extracted from concrete elements, simulating the same work conditions of the applied concrete in the real structure. Based on the obtained results, it is possible to observe that:

- Generally, self-compacting concrete presented better durability indicators than the conventional vibrated concrete. This result may contribute to elucidate the reliability of the SCC application in more aggressive environments. This way, it is demonstrated that using SCC instead of CC of similar composition may contribute to higher durability of structures.
- The electrical resistivity experiment showed that SCC result has a despicable rate of probable corrosion to 28 and 90 days, with absolute resistivity values superiors to the CC ones. Additionally, it was verified that resistivity is heavily influenced by the presence of humidity in porous concrete. So, it seems that SCC demonstrated to be more durable than CC.
- The chlorides diffusion experiment classified the probable chloride penetration as very low in the SCC and low in the CC, at both 28 and 90 days. This way, the chloride diffusion experiment confirms the resistivity experiment, and shows that SCC seems to be more durable than CC.
- In the water absorption through capillarity experiment it was demonstrated that SCC and CC performances were similar.
- When testing voids volume, SCC presented lower values than CC, which represents a higher durability performance.
- In the accelerated carbonation test, at both 28 and 90 days, the values for the carbonation front obtained were higher for the CC in relation to the SCC. However, it was found that the carbonation front for the SCC and CC was superior for 90 days, due to the higher moisture content in the concrete at 28 days, making CO_2_ entrance difficult. So, the SCC presented itself a better durability indicator than the CC.
- Higher porosity in CC than in SCC was observed in both scanning electron microscope and micro tomography tests. When the porous volume results of both concretes were verified, through micro tomography tests, it was noted that the relation between the porous volume and the VOI is 80% higher in the CC than in the SCC.
